# Real-time automatic detection of starch particles in ambient air

**DOI:** 10.1016/j.agrformet.2022.109034

**Published:** 2022-08-15

**Authors:** Branko Šikoparija, Predrag Matavulj, Gordan Mimić, Matt Smith, Łukasz Grewling, Zorica Podraščanin

**Affiliations:** a BioSensе Institute-Research Institute for Information Technologies in Biosystems, University of Novi Sad, Dr Zorana Djindjica 1, Novi Sad 21000, Serbia; b School of Science and the Environment, University of Worcester, UK; c Laboratory of Aerobiology, Department of Systematic and Environmental Botany, Adam Mickiewicz University, Poznań, Poland; d Department of Physics, Faculty of Sciences, University of Novi Sad, Novi Sad, Serbia

**Keywords:** Aerobiology, Airborne starch, Automatic monitoring, Dispersion modelling, Emission sources

## Abstract

Considerable amounts of starch granules can be present in the atmosphere from both natural and anthropogenic sources. The aim of this study is to investigate the variability and potential origin of starch granules in ambient air recorded at six cities situated in a region with dominantly agricultural land use. This is achieved by using a combination of laser spectroscopy bioaerosol measurements with 1 min temporal resolution, traditional volumetric Hirst type bioaerosol sampling and atmospheric modelling. The analysis of wind roses identified potential sources of airborne starch (i.e., cereal grain storage facilities) in the vicinity of all aerobiological stations analysed in this study. The analysis of the CALPUFF dispersion model confirmed that emission of dust from the location of storage towers situated about 2.5 km north of the aerobiological station in Novi Sad is a plausible source of high airborne concentrations of starch granules. This study is important for environmental health since it contributes body of knowledge about sources, emission, and dispersion of airborne starch, known to be involved in phenomena such as thunderstorm-triggered asthma. The presented approach integrates monitoring and modelling, and provides a roadmap for examining a variety of bioaerosols previously considered to be outside the scope of traditional aerobiological measurements.

## Highlights

• Quantification of starch in ambient air by laser spectroscopy bioaerosol monitoring.
• Insights into atmospheric processes via high temporal resolution data.
• Identification of sources of airborne particles.
• Notable amounts of airborne starch are related to cereal grain storage facilities.

## Graphical abstract

**Figure:**
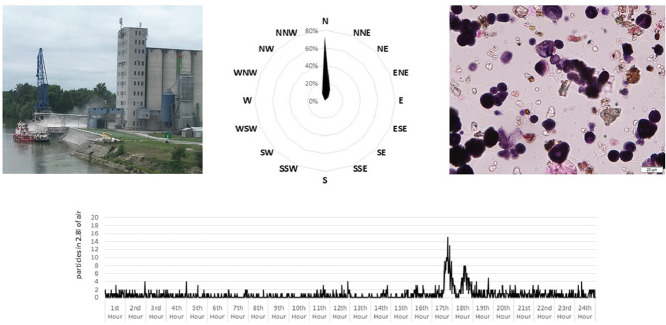

## 1 Introduction

There is a wide spectrum of biological material suspended in the atmosphere (i.e. living organisms and their fragments and products) (Šantl-Temkiv et al., 2020), and the diversity and quantity of this material depends on the source characteristics and atmospheric conditions. The impact bioaerosols have on the environment and living organisms puts them at the forefront of many research fields, from atmospheric physics and air quality to plant protection and occupational and public health. Standard methods of air quality monitoring only provide information about the concentrations of size fractions of bioaerosols in ambient air (e.g. PM2.5, PM10), which is not enough to assess their diversity or address their impact.

Interest in bioaerosols underpins aerobiology, a multidisciplinary field of study where practitioners identify and quantify airborne particles of biological origin and examine the effects of such particles on living systems and on the environment. Networks of aerobiological stations have been set up across the world for the long-term monitoring of important outdoor aeroallergens (Scheifinger et al., 2013; Buters et al., 2018) and plant pathogens (Isard et al., 2011; Neufeld et al., 2018), and a standardised method EN16868:2019 has been established for the sampling and analysis of airborne pollen grains and fungal spores (CEN, 2019). Due to advances in sampling and analysis techniques, aerobiologists have shifted their focus away from simply examining organisms and cells and have broadened their horizons to encompass airborne molecules like allergenic proteins (Buters et al., 2012; Galan et al., 2013; Grewling et al., 2016, 2020).

Starch is a polysaccharide produced by plants and serves as energy storage. It is synthesized in the form of granules that are insoluble in water at room temperature (Jane, 2009). The morphology of granules is characteristic of the botanical source and their size can vary from submicrometric to more than 100 µm (Jane et al., 1994). Plants accumulate starch where energy is needed, such as in seeds, fruits, roots and tubers, but starch granules can also be in pollen grains where they indicate evolution patterns regarding the size of pollen (i.e. starchy grains being larger) and pollination strategy (i.e. entomophilous pollen are oil rich while anemophilous are predominately starchy) (Baker and Baker, 1979). Starch granules can be released and become airborne from plant cells, through the apertures of pollen, or when pollen grains rupture (Laurence et al., 2011 and references therein). Considerable amounts of starch granules can be present in the atmosphere from natural processes. Laurence et al. (2011) used the term “starch rain” when describing significant contamination of archaeological artifacts by airborne starch. Moreover, exposure to starch granules released by osmotic shock from pollen grains during thunderstorms could impact environmental health in the form of thunderstorm-triggered asthma (Newson et al., 1997; Harun et al., 2019 and references therein).

On the other hand, anthropogenic activity, such as the production and processing of cereal grains for human and animal use (i.e., transport, storage, drying, loading, milling), can result in large emissions of airborne dust (McLouth and Paulus, 1961; Stobnicka and Górny, 2015) that contains more than 50% starch (Schanke, 1981). Dust from grain handling can be hazardous because its high organic content and physical properties (Boac et al., 2009) makes it explosive (US EPA, 2003). In addition, experimental studies have shown that exposure to airborne starch results in subclinical inflammation of the airways and an accumulation of eosinophils (Grunewald et al., 2003). Starch granules emitted from grain handling processes contain a number of bioactive substances (Stobnicka and Górny, 2015), such as bioactive remains of plant cells on the surface that if inhaled can cause baker's asthma (Stobnicka and Górny, 2015), one of the most severe and frequent manifestations of occupational allergy (Skjold et al., 2008; Page et al., 2010).

New aerobiological methods developed to increase the specificity of detections, e.g. identification based on molecular analysis (Kraaijeveld et al., 2014), or to enable measurements in real-time at high temporal resolution (Tesendic et al., 2020), provide opportunities to study bioaerosols that have not previously been examined in detail even though they are known to be present in aerobiological samples. The aim of this study is to investigate the variability and potential origin of starch granules in ambient air. This is achieved by using a combination of laser spectroscopy analysis of single aerosol particles, volumetric Hirst type bioaerosol sampling and atmospheric modelling.

## 2 Material and methods

### 2.1 Study location

**Fig. 1:**
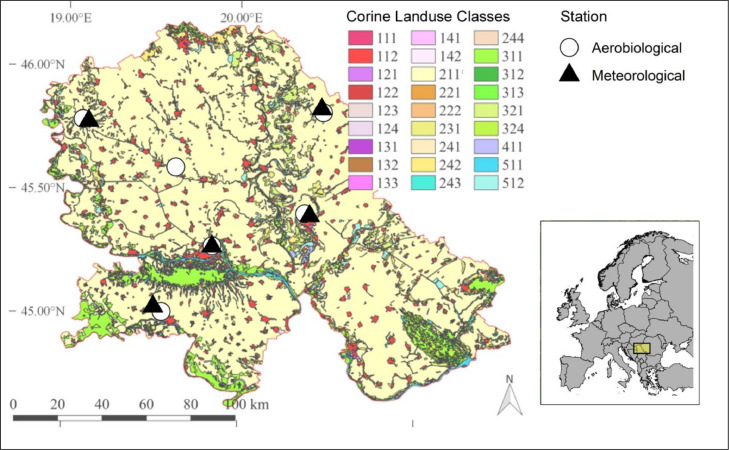
Study location with the location of aerobiological and wind direction measurements.

The study was conducted in the Autonomous Province Vojvodina (Serbia), located on the southern part of the Pannonian Plain, from 16 February to 6 October 2019. The Pannonian Plain is a region predominantly occupied by agriculture, particularly arable crops (Lugonja et al., 2019). There is a long history of intensive wheat and corn production in Vojvodina and these crops occupy more than 60% of land under arable cultivation (Statistical Office of the Republic of Serbia, 2020). As a result, there are many grain processing facilities across the region. Bioaerosol measurements were performed at the roof level at six locations: Kikinda, Novi Sad, Sombor, Sremska Mitrovica, Zrenjanin and Vrbas, and representative wind direction was recorded at five of these locations (Fig. 1, Table 1).

**Table 1:** Location of aerobiological and meteorological stations used in the study.

Station name	Aerobiological station	Meteorological station	
	Latitude / Longitude	Index number	Latitude / Longitude
Novi Sad	45.25 / 19.85	-	45.25 / 19.85
Kikinda	45.83 / 20.48	13174	45.85 / 20.47
Sombor	45.78 / 19.11	13160	45.77 / 19.15
Sremska Mitrovica	44.97 / 19.61	13266	45.01 / 19.55
Zrenjanin	45.38 / 20.39	13173	45.37 / 20.42
Vrbas	45.57 / 19.64	-	-

### 2.2 Bioaerosol measurements

#### 2.2.1 Volumetric Hirst method

The Hirst type volumetric trap (Hirst, 1952) is the standard method used for the aerobiological monitoring in Europe and many other parts of the world (Buters et al., 2018; CEN, 2019). It allows both qualitative and quantitative analysis of airborne pollen and fungal spores but also other bioaerosols. We sampled air by using Hirst type Lanzoni VPPS2000 impaction samplers that continuously draw in 10 L min^−1^ of air through an orifice oriented towards the direction of the wind. The particles suspended in the atmosphere are impacted onto an adhesive tape that is mounted on a drum that rotates at 2 mm/h behind the 2 mm  ×  14 mm orifice. A 48 mm section of tape corresponding to 24-h sample is then mounted on a microscope slide and analysed under the light microscope at x400 magnification. The slides are scanned at horizontal transects and bioaerosols of interest are recorded and quantified at 2 mm segments, which corresponds to hourly samples (Sikoparija et al., 2018).

**Fig. 2:**
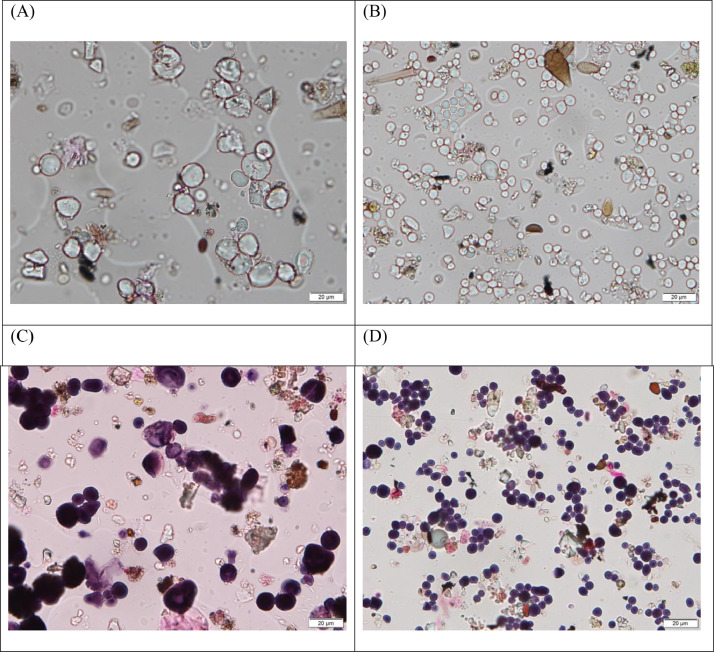
Starch granules adhered to sampling surface of Hirst type volumetric sampler (EN16868:2019) in Novi Sad on 27 February 2019 (A and C) and in Zrenjanin 7 August 2019 (B and D). Lower images depict starch granules in the same samples after staining using Lugol's iodine. Micrographs are taken at x400 magnification using Olympus BX51 upright microscope with the Olympus DP26 camera.

The routine analysis of microscope slides revealed periods containing notable numbers of granules (Fig. 2A and B), which we suspected to be starch based on their morphology (Chakraborty et al., 2018). To confirm this, we selected two slides containing granules, one from Novi Sad and the other from Zrenjanin, removed the cover slips and rinsed the sample surfaces with Lugol's iodine I2-IK (Cortella and Pochettino, 1994). Samples were mounted with coverslips using glycerine-jelly and the granules inspected for colour change. Change to dark blue (Fig. 2C and D) confirmed our hypothesis that the detected particles were starch granules.

In order to examine characteristics of airborne starch granules in the studied region, all daily samples collected in 2019 were reanalysed by light microscopy to identify the hours when large numbers of starch granules were present. Thirteen daily slides from Novi Sad that contained notable amounts of starch granules were selected for further quantitative analysis; starch granules > 8 μm were counted along three longitudinal transects at x400 magnification and data were expressed as concentration (starch granules m^−3^). These data were used for validating measurements by real-time bioaerosol monitor.

#### 2.2.2 Automatic bioaerosol measurements

The Rapid-E instrument (Plair SA) is a laser spectroscopy-based aerosol monitor. It continuously samples 2.8 L min^−1^ of air through a fixed two-layered Sigma-2 inlet (VDI 2119, 2013). Each sampled particle, while carried by the air stream, interacts with the laser light sources resulting in scattered light and fluorescence (Šaulienė et al., 2019). The collected signal is suitable for the identification of bioaerosols as it provides information about their morphology and chemical composition (Kiselev et al., 2013).

**Fig. 3:**
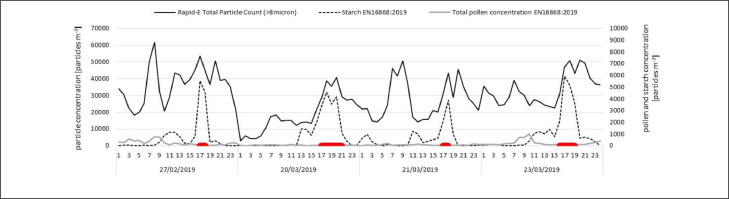
Total particle concentrations measured by Rapid-E bioaerosol monitor, starch particle concentrations and total pollen concentrations measured by Lanzoni VPPS2000 (EN16868:2019). Periods from which particles were taken for training the artificial intelligence classification model are indicated by horizontal lines.

The Rapid-E was installed in Novi Sad next to a Lanzoni VPPS2000 sampler and operated in the “smart pollen mode” (Sikoparija, 2020) which quantifies the number of particles > 8 μm in optical diameter. The preliminary analysis indicated that the total count of particles > 8 μm alone was not enough to identify starch (Fig. 3). Therefore, we have used deep learning to classify starch granules from other > 8 μm particles detected per minute by Rapid-E bioaerosol monitor (Šaulienė et al., 2019; Tesendic et al., 2020).

The classification algorithm is based on multi-input one-output convolutional neural network (CNN) that allowed combining multiple inputs from the Rapid-E device to perform classification. As described in Tesendic et al. (2020), the network processes each input type with convolutional blocks specific for the input, which contain a set of 2D convolutions, batch normalization layers, ReLU activation functions, maxpool and dropout. The features obtained at the final convolutional blocks are then concatenated across the inputs and the classification is performed. Ninety percent of samples labelled as starch were used for training CNN while the remaining events were used for validation. Rapid-E measurement data from four periods were used for training and validating a deep learning classification model. These periods were selected based on large quantities of starch granules being detected and very few other large bioaerosols being present. A total of 982 representative particles were recorded during 2019, on 27 February (16:47-18:15UTC), 20 March (17:01-17:42UTC, 18:01-18:06UTC, 19:01-19:58UTC), 21 March (17:07-17:23UTC) and 23 March (16:37-17:43UTC) and labelled as starch granules (Fig. 3). The performance of automatic detections was tested by comparing Rapid-E data to hourly starch concentrations obtained by standard volumetric Hirst method - EN16868:2019 for ten days when large quantities of starch were detected in Novi Sad, but which were not used in training the model.

### 2.3 Atmospheric conditions and transport

#### 2.3.1 Meteorological measurements

Wind directions were analysed to search for the sources of the starch granules recorded at aerobiological stations. For four locations, Kikinda, Sombor, Sremska Mitrovica and Zrenjanin, we extracted wind data from automated weather stations belonging to the official observation network operated by the Hydrometeorological Service of Serbia and situated in vicinity of aerobiological stations (Table 1). At the Novi Sad station, the wind angle in cartesian coordinating system was obtained in degrees, from components of the wind speed *u* and *v* (in *x* and *y* direction, respectively) measured at 10 Hz by using 3D ultrasonic anemometer (Young 81000 from Campbell Scientific Inc.) installed besides the bioaerosol samplers. The conversion was performed to calculate the meteorological angle representing wind direction, having in mind that 0° corresponds to the wind from the north. There is no representative meteorological station in Vrbas, and so we were unable to perform wind direction analysis for that location.

#### 2.3.2 Dispersion modelling

The Lagrangian puff model CALPUFF (Scire et al., 2000a) has been used to confirm whether starch particles emitted from suspected sources can be brought by air masses to the aerobiological measurement station in Novi Sad. The outputs from WRF 3.6.1 (Weather Research and Forecasting Model) were used as necessary meteorological fields in the CALPUFF model. The WRF model version 3.6.1 with horizontal grid size of 10 km and 36 vertical levels were used in simulations. The model domain covered 151 × 151 grid points with the centre at 45.25 N and 9.85 E. The RRTM (Rapid Radiative Transfer Model) longwave radiative scheme (Mlawer et al., 1997), Dudhia shortwave radiative scheme (Dudhia, 1989), WSM3 microphysics (Hong et al., 2004), Noah land-surface model (Tewari et al., 2004), YSU PBL physics scheme (Hong et al., 2006) and Kain-Fritch cumulative schemes (Kain and Fritsch, 1993; Kain, 2004) were used in simulations. The simulations were initialized with GFS (Global Forecast System) forecast data with horizontal resolution of 0.25 degrees. To reduce meteorological grid space, the CALMET model was run using the WRF outputs (Scire et al., 2000b). The CALPUFF model was run with 50 m horizontal grid size (301 × 301 grid points) and 10 vertical levels. We assumed that the starch granules are emitted from point sources at 1.5 m with 1 m radius, and continuous emission rate of 1 g s^−1^ and 18 m s^−1^ velocity. The geometric mass mean diameter was 10 μm and the geometric standard deviation was 4 μm.

## 3 Results

### 3.1 Airborne starch detections across the studied region in 2019

**Fig. 4:**
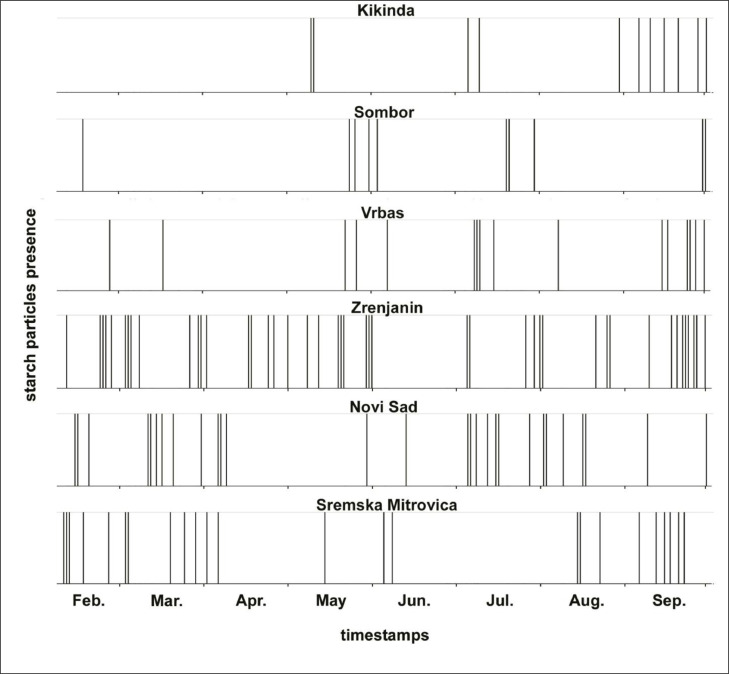
Detections of starch granules in aerobiological stations across study region in 2019. Each vertical line indicate hour when large quantity of starch is registered on microscopic slides with aerosol samples collected using EN16868:2019.

The analysis of aerobiological samples detected only a few clusters of starch granules packed within plant material, but numerous episodes when isolated starch granules were predominant. Temporal distribution of starch detections during 2019 is shown in Fig. 4. There were no discernible patterns and no obvious relation between detections at different sites, suggesting that detections were intermittent and affected by local factors only. The occurrence of starch was more frequent at southern stations, i.e., Sremska Mitrovica, Novi Sad and Zrenjanin. There was no notable regularity with respect to day of week (Supplementary Fig. S1) and starch was detected both on workdays and during weekends. Analysis showed that although starch particles are detected in all periods of the day there is a tendency towards more records from 16-22h UTC (Supplementary Fig. S2).

### 3.2 Detection of airborne starch at high temporal resolution

Overall accuracy of the artificial intelligence classification model for the identification of 26 classes (24 pollen classes, a class representing starch and a class representing spores and other non-pollen biological particles) was 65.3% for the validation dataset. Starch granules are identified with 82% accuracy, mostly being confused with *Broussonetia* and *Ulmus* pollen while a small percentage (∼5%) of *Broussonetia, Morus, Platanus* and *Poaceae* are misclassified as starch.

**Fig. 5:**
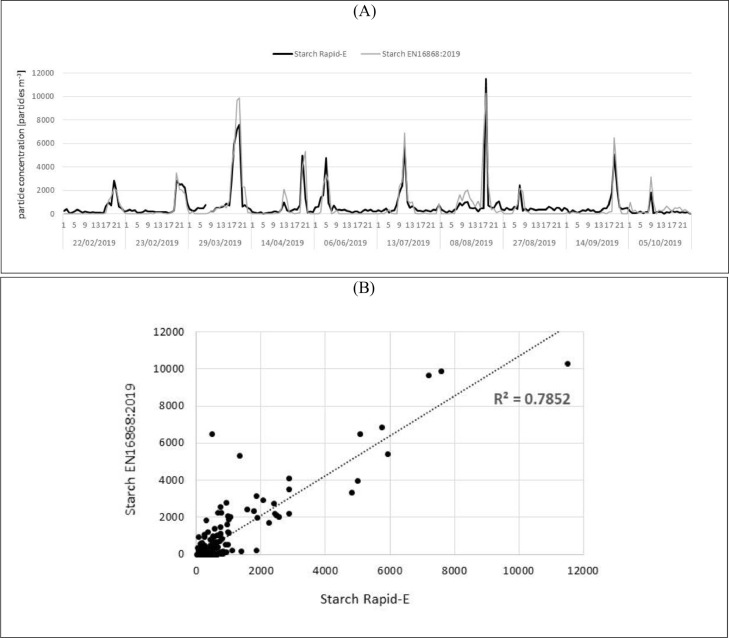
Accuracy of starch detections using Rapid-E bioaerosol monitor when comparing to EN16868:2019; (A) timeseries of starch granules concentrations and (B) respective scatter diagram with calculated coefficient of determination. (Rapid-E concentrations were scaled linearly with the scaling factor 4.53 obtained by dividing the sum of hourly starch concentrations recorded with EN16868:2019 by the sum of hourly starch concentrations recorded using Rapid-E bioaerosol monitor from four days when data was used for training the classification model).

A highly significant positive Pearson correlation coefficient (r = 0.87, p<0.01) confirmed satisfactory performance of the airborne starch detections using the Rapid-E device, which allowed us to use 1 min resolution data during further analysis (Fig. 5).

Measurements of airborne starch at 1 min temporal resolution reveal that recorded starch episodes are often shorter than an hour (Supplementary Fig. S3) reaching up to 7143 starch granules m^−3^. Sudden increases in airborne starch are usually followed by sudden decreases. However, starch is recorded more frequently and with a notable quantity, during the minutes following the peak. The 1 min records are further explored to identify potential sources.

### 3.3 Origin of airborne starch

**Fig. 6:**
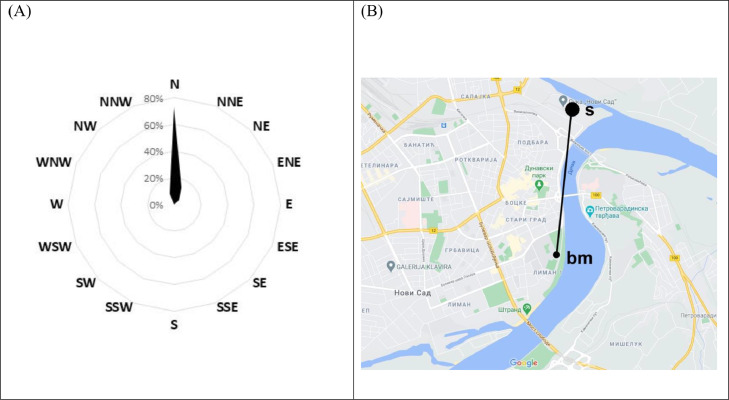
(A) Wind rose for 22 h when the most starch granules were recorded in Novi Sad, (B) location of potential source(s) of airborne starch, i.e. port and storage towers, in relation to bioaerosols measuring station (bm) as displayed in Google Maps (https://www.google.com/), line indicates distance of about 2.5 km as measured by Google Earth (https://earth.google.com/).

Average hourly wind directions during hours with the highest number of starch granules recorded in Novi Sad indicated potential sources towards the north (Fig. 6A). Northerly winds were predominant for all periods when the Rapid-E bioaerosol monitor detected > 2 starch particles (Supplementary Fig. S4). At approximately 2 km to the north from the bioaerosol measurement station there is a port and towers for storage and processing cereals (Fig. 6B). Since unloading can generate large clouds of flour (Supplementary Fig. S5) we speculate that storage towers are the major source of airborne starch in the study region.

A GoogleEarth search (https://earth.google.com/) confirmed that, there are storage towers in the vicinity of each aerobiological station, i.e., Kikinda (0.8 km to the NNE and 1.5 km to the S), Sombor (4.5 km to the SSW-SW and 5 km to the SSE-SE), Sremska Mitrovica (1.3 km to the NNE and 2.8 km to the SE-ESE), Vrbas (1.3 km to the NW) and Zrenjanin (1.3 km to the SW-SSW and 2.7 km to the S-SSE) (Supplementary Fig. S6). Predominant wind directions at the specific station during high starch records were: WNW, N-NNE, S-SSE in Kikinda, SSE-ESE in Sombor, E-ESE in Sremska Mitrovica and SSE in Zrenjanin (Supplementary Fig. S6).

**Fig. 7:**
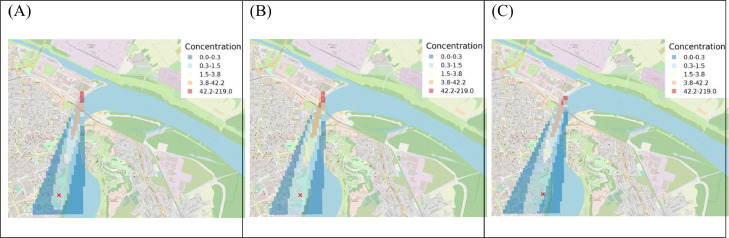
CALPUFF simulation of particles dispersion emitted from location of storage towers situated at about 2.5 km distance from the bioaerosols measuring station in Novi Sad on 22 February 2019 at (A) 20h UTC, (B) 21h UTC and (C) 22h UTC. “X” indicates location of bioaerosol measurements.

Analysis using the CALPUFF model of a selected starch episode (Fig. 7) confirmed emission of dust from the location of storage towers situated at about 2.5 km north of the aerobiological station in Novi Sad is a plausible source of high airborne concentrations of starch granules.

## 4 Discussion

The presence of isolated starch granules in aerobiological samples is in line with findings from Laurence et al. (2011). The large size (> 8 μm) of detected starch granules indicates they originate from storage organs rather than pollen. Starch, when released by pollen germination or the rupture of the exine, is detected in the fraction < 8 μm (Suphioglu et al., 1992; Schäppi et al., 1999). In addition, we have detected notable quantities of airborne starch in February and September, which is outside the period when pollen is present in the atmosphere.

Despite minor uncertainty, the automatic particle identification, using a combination of laser spectroscopy signal and artificial intelligence, successfully discriminated airborne starch from particles in the same size range. Misidentification with pollen is understandable and can be attributed to uncertainty of the training dataset, which was obtained from measurements coinciding with the occurrence of misidentified pollen types in the atmosphere (Tesendic et al., 2020). Earlier studies confirmed the possibility of classifying starch types using flow cytometry (Zhang et al., 2018), but requirements to stain samples and analysis in liquid limits its use for real-time detection and quantification of airborne starch granules.

Although a few small sources present in the region (i.e. bakeries) could contribute to atmospheric concentrations of starch, the analysis of wind roses identified large sources of airborne starch in the vicinity of all aerobiological stations analysed in this study (Supplementary Fig. S6). We can see in the case of Sombor (Fig. S6B) and Zrenjanin (Fig. S6D) that wind roses did not implicate large potential sources of airborne starch to the WSW-SW as being responsible for intensive starch episodes, which suggests lower activity in those facilities at the time. This supports our hypothesis that starch emission is intermittent and depends on anthropogenic activity (i.e. processing grain), and the records of starch at the monitoring station depend on aligning the wind direction with emission processes. Although it is expected that the frequency of starch detections is going to be larger at sites closer to storage towers, more sources surrounding the site in different directions, like in Sremska Mitrovica, increase the odds that wind direction is suitable for transport and thus could result in more frequent detections. It should be noted that starch granules recorded in Zrenjanin were of notably smaller size, making them more susceptible for dispersion under a wider range of wind conditions.

The CALPUFF dispersion model was used to investigate the possibility that starch granules emitted from the suspected source in Novi Sad (i.e. port and towers for the storage of cereals) could be carried by northerly winds to reach the measurement station. For detailed analysis and comparison of the modelled and measured starch concentration, precise determination of the emission rate and time, source height, radius and diameter are required. Also, more details about the meteorological conditions (i.e. vertical wind speed and temperature) should be combined with the WRF model. Finally, it should be noted that the study area is complex with respect to terrain and for more precise quantitative assessment of particle dispersion buildings should be added to the CALPUFF model.

Based on the knowledge that the density of starch is 1.5 g cm^−3^ (Jay-Lin 2009), and assuming starch particles are spherical 10 μm in diameter, we can calculate the mass concentration of starch suspended in the atmosphere. The quantity of starch measured per hour on selected days in Novi Sad reached over 10000 starch granules m^−3^ which corresponds to about 0.008 mg m^−3^. Knowing that the peaks are usually recorded over shorter periods of time (Supplementary Fig. 3) the exposure concentration is even higher (e.g. maximum recorded 1 min concentration of starch granules was 0.025 mg m^−3^ on 29 March). In our study, we have quantified starch granules only as particles > 8 μm. However, smaller fractions were also present in our samples, which suggests the present study underestimates the total amount of airborne starch and its mass concentrations per hour. Environmental challenge chamber tests, with hourly concentrations lower than described in this study, showed that starch granules can trigger inflammation of the lower airways (Badorrek et al., 2012). Occupational exposures to high concentrations of flour dust are frequently observed but are usually of a short duration under four minutes (Stobnicka and Górny, 2015), while the episodes we identified lasted longer. The levels of airborne starch detected in this study, although below exposure levels seen in indoor environments where flour is manipulated, could potentially impact environmental health by reaching occupational exposure limits (1 mg m^−3^) specified by the Scientific Committee on Occupational Exposure Limits (Scientific Committee on Occupational Exposure Limits SCOEL, 2008). In addition, it should be noted that the concentration of airborne starch granules will be notably higher nearer to the point of origin and so exposure is expected to be greater for those living near the source. Therefore, in areas where a few potent sources of airborne starch are present (i.e. storage towers), starch can become an environmental health hazard.

The quantity of starch granules detected here exceeded estimated quantities of airborne starch that could originate from airborne pollen, i.e. about 700 per pollen grain (Schäppi et al. 1999). Although, the number of starch granules exuded from pollen grains has been shown to significantly increase on days when rainfall is recorded (Suphioglu et al., 1992; Schäppi et al., 1999). Several authors have implicated starch granules released from pollen in asthma exacerbations related to thunderstorms (Knox 1993, Newson et al., 1997; Atkinkinson and Strachan, 2004; Harun et al., 2019). Starch granules from pollen are small enough to reach the lower airway and can contain allergens (Suphioglu et al., 1992; Harun et al., 2019), but it depends on the protein involved. For instance, Suphioglu et al. (1992) and Taylor et al. (1994) identified the major allergen Lol p 9 in the starch granules (amyloplasts) of *Lolium perenne* pollen grains. Whereas Taylor et al. (1994) found that starch from *L. perenne* pollen was only lightly labelled by Lol p 1 and Grote et al. (2000) found expelled starch granules to be free from Lol p 1 and Lol p 5. Similarly, when examining the major allergens of *Phleum pratense* Grote et al. (1994) did not find Phl p 1 in starch granules but Phl p 5 was present. Nevertheless, starch granules could act as allergen carriers as allergen-containing subcellular debris can be attached to the surface of starch granules (Grote et al., 2000).

In addition, starch produced from contaminated stored cereals can contain a notable amount of aflatoxins (Aly, 2002) which are strong carcinogens that pose an important occupational health risk by causing primary liver and respiratory cancers (Tang et al., 2019 and references therein). Although ingestion is the dominant exposure pathway for the general population, in areas like the Pannonian part of Serbia, where high aflatoxin levels could be accumulated due to prolonged drought conditions (Kos et al., 2013) and notable amounts of airborne starch can be emitted from grain processing facilities, the estimated daily intake levels could be underestimated if risk assessment studies rely only on dietary exposure (Udovicki et al., 2021).

As we have seen in the case of airborne starch, high resolution automatic bioaerosol detection can provide warnings in real-time of the negative effects of anthropogenic activity and thus support mitigation efforts for protecting public health. Furthermore, 1 min resolution data can give insights into natural phenomena such as thunderstorms and, therefore, greater understanding of the role of airborne particles of biological origin, such as starch and aeroallergens, in asthma exacerbations (Atkinkinson and Strachan, 2004). The results presented emphasize the importance of new approaches in detecting bioaerosols. The availability of such high-resolution data will provide valuable knowledge about acute (i.e. short-term, high magnitude) exposure that can significantly increase the risks of allergic and asthmatic symptoms (Kitinoja et al., 2020). Note that Kitinoja et al. (2020) defined short-term as 1-day and so more work is needed to determine the impact of exposure over even shorter time scales.

Starch granules are only one of many "neglected" biological particles, which may have significance for animal and human health and the environment. For instance, real-time automatic bioaerosol samplers will allow the detection of many potent airborne pathogens, such as fungal spores, which are often not routinely monitored due to morphological similarities between taxa. On the other hand, the analysis by light microscope requires trained specialists (Galán et al., 2021), which is extremely time consuming and expensive in terms of staffing hours. In theory, automated methods could also be used to detect much rarer but still biologically important particles, e.g. airborne cyanobacteria and algae (Sherwood et al., 2020; Wiśniewska et al., 2020), testate amoebae (Wanner et al., 2015) or tardigrade propagules (Pugh and McInnes, 1998). This could provide information about processes involved in ecological colonisation, e.g. in the case of testate amoebae (Wanner et al., 2015).

## 5 Conclusion

In this study, we have investigated the variability and potential origin of starch granules in ambient air and provided a roadmap for examining a variety of bioaerosols previously considered to be outside the scope of traditional aerobiological measurements. The advantages of automatic techniques for bioaerosol detection over traditional monitoring can be clearly seen. Laser spectroscopy-based automatic bioaerosol measurements equipped with machine learning algorithms enable the identification and quantification in high temporal resolution of airborne particles of biological origin, such as starch granules, that are often overlooked during routine microscopic analysis. With proper training datasets and dense automatic monitoring networks, the spatiotemporal distribution of these neglected biological particles could be obtained. Such data can help answer crucial evolutionary questions about colonization processes and species diversity on earth.

## Declaration of Competing Interest

The authors declare that they have no known competing financial interests or personal relationships that could have appeared to influence the work reported in this paper.

## Appendix Supplementary materials

Image, application 1
