# Supplementary material for: Real-time automatic detection of starch particles in ambient air
Source: Agric For Meteorol. 2022 Aug 15;323:109034. doi: 10.1016/j.agrformet.2022.109034 (PMC9391928; doi:10.1016/j.agrformet.2022.109034)
Supplement: Supplementary file 1 [file mmc1.doc]

**Real-time automatic detection of starch particles in ambient air**

| 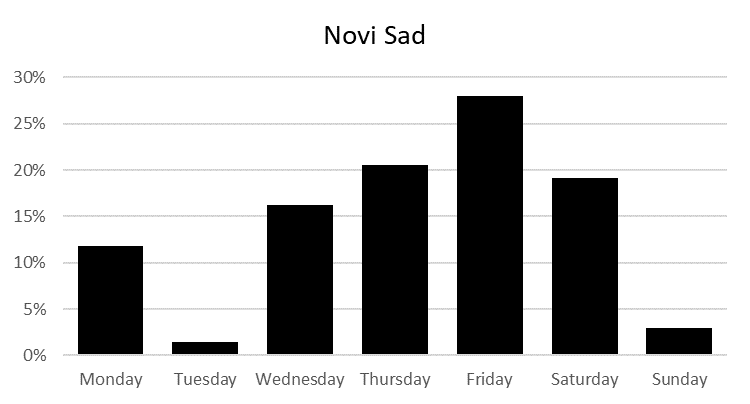 |
| --- |
| 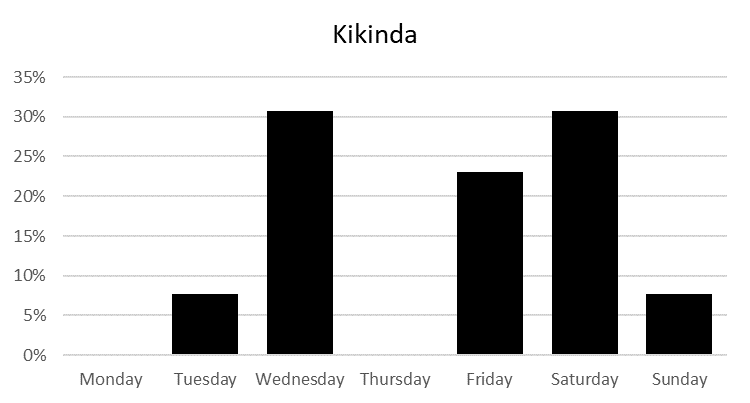 |
| 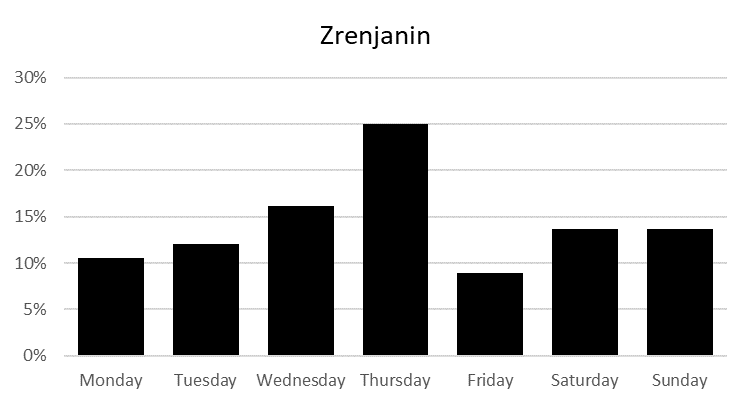 |
| 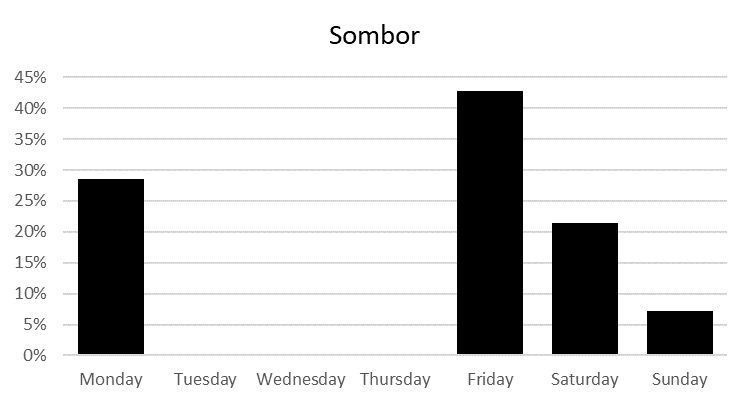 |
| 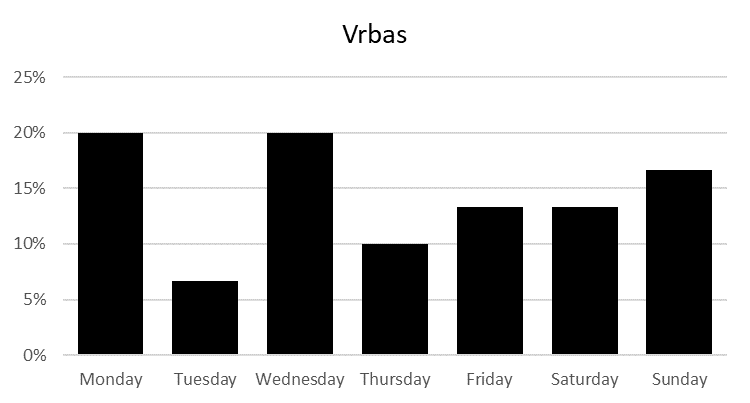 |
| 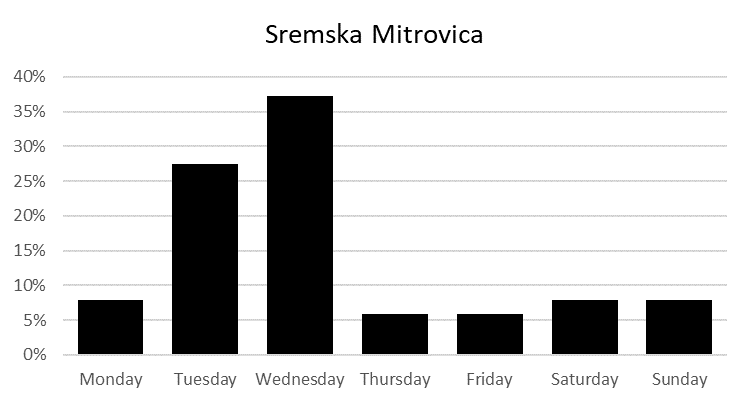 |
| Supplementary Figure S1 Percentage contribution of starch episodes across the days of the week over studied region in 2019 |

| 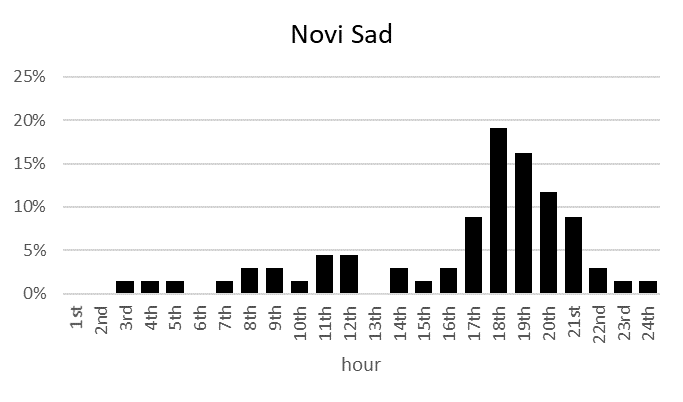 |
| --- |
| 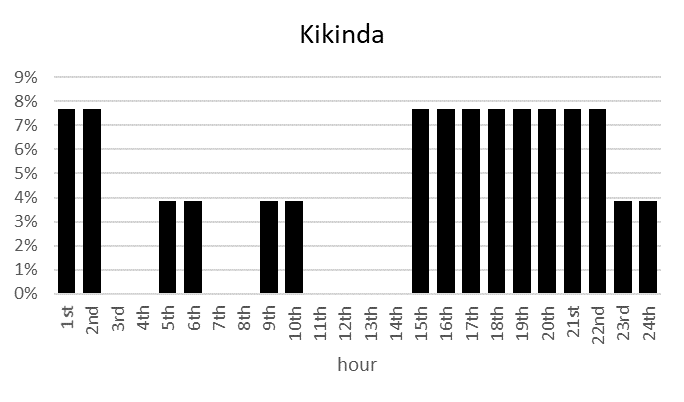 |
| 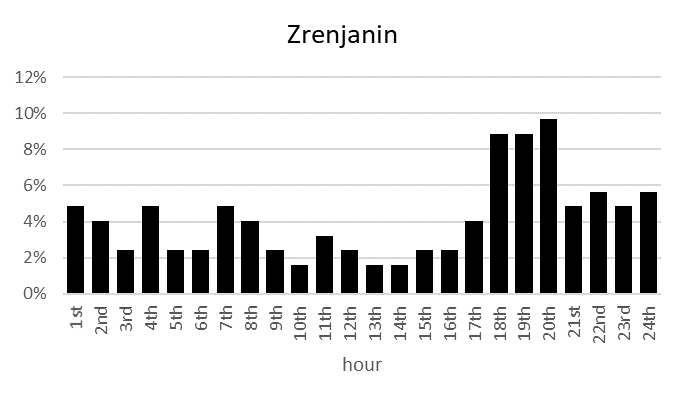 |
| 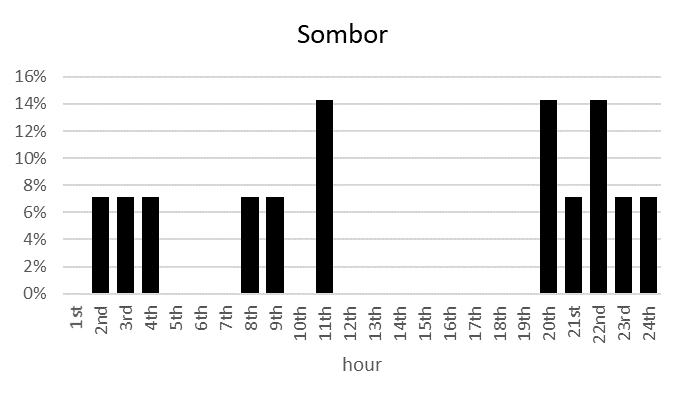 |
| 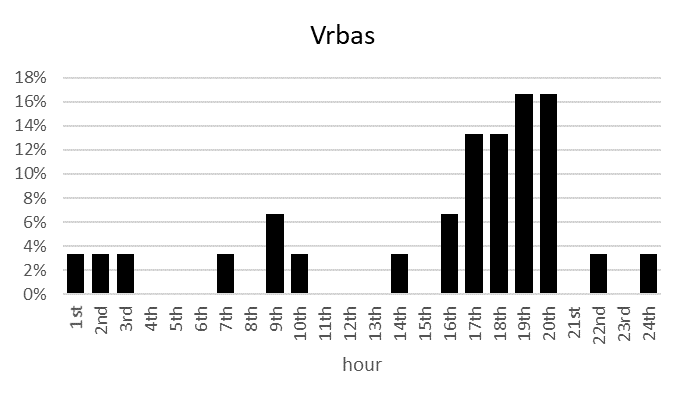 |
| 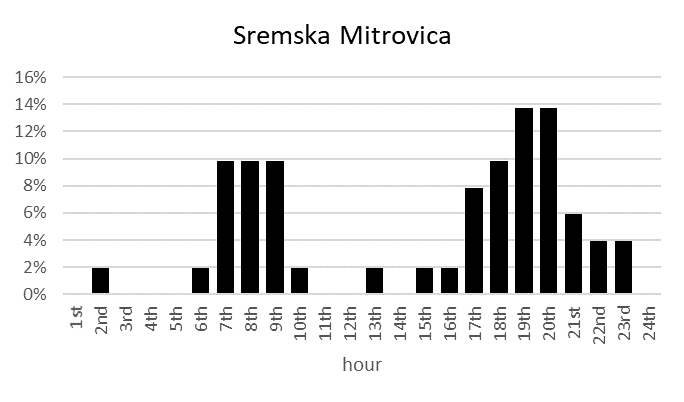 |
| Supplementary Figure S2 Percentage contribution of starch episodes across UTC hours of the day over studied region in 2019 |

| *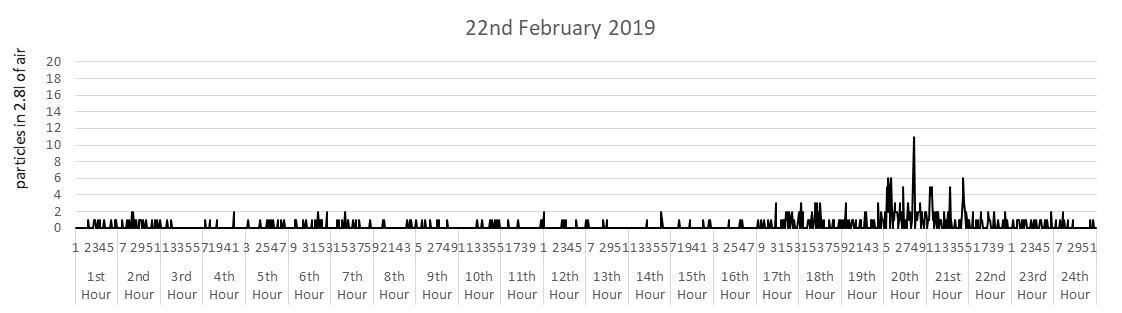* |
| --- |
| *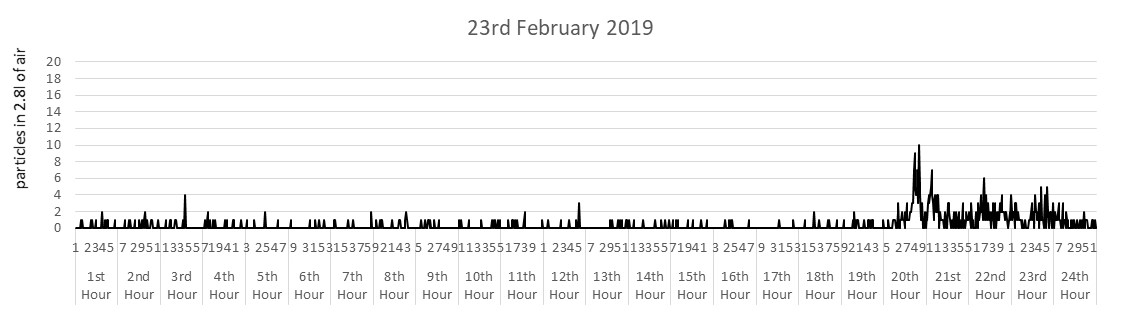* |
| *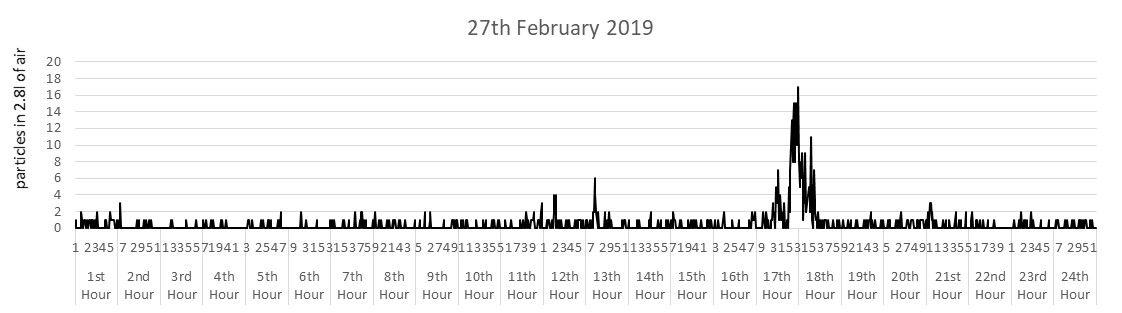* |
| *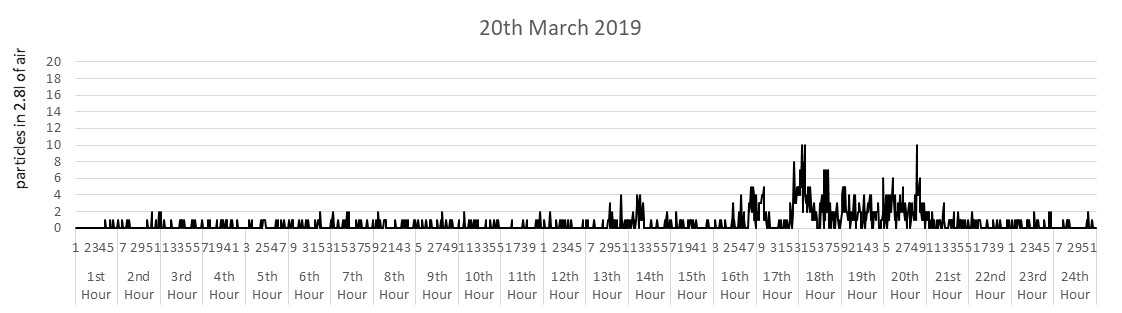* |
| *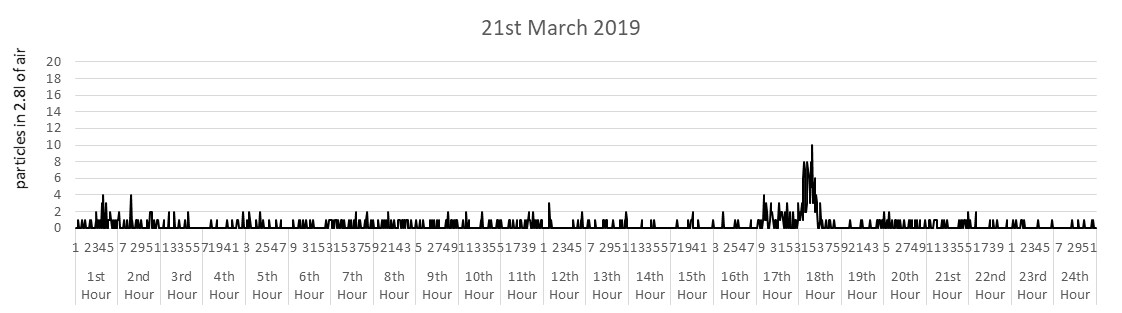* |
| *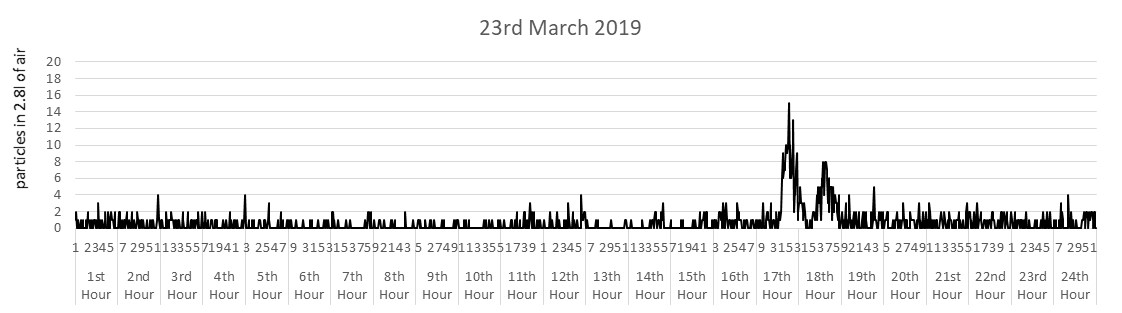* |
| 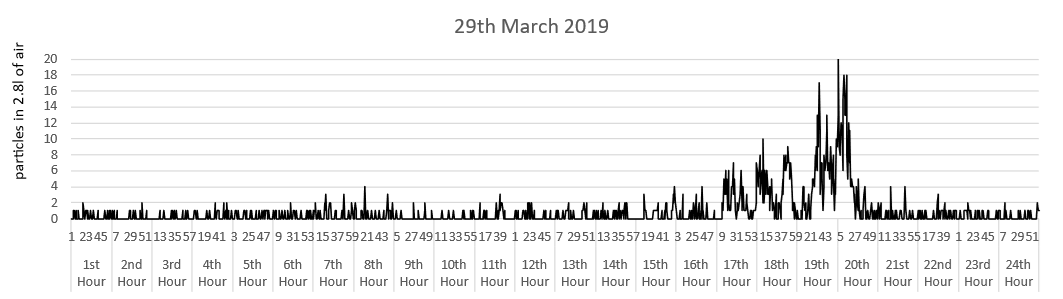 |
| *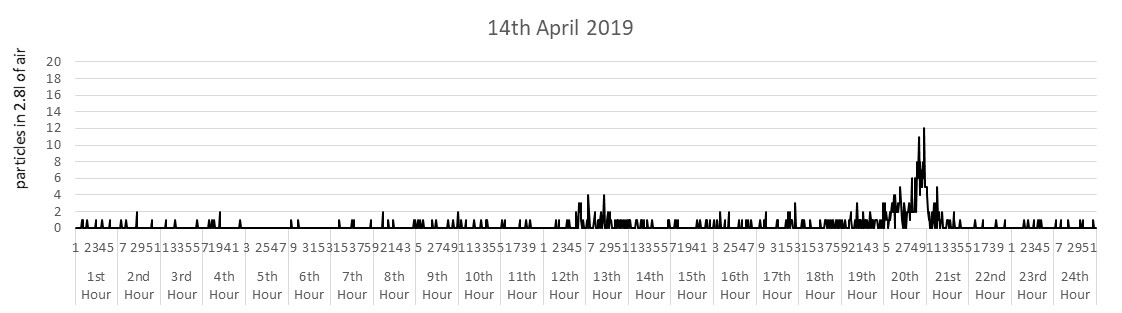* |
| *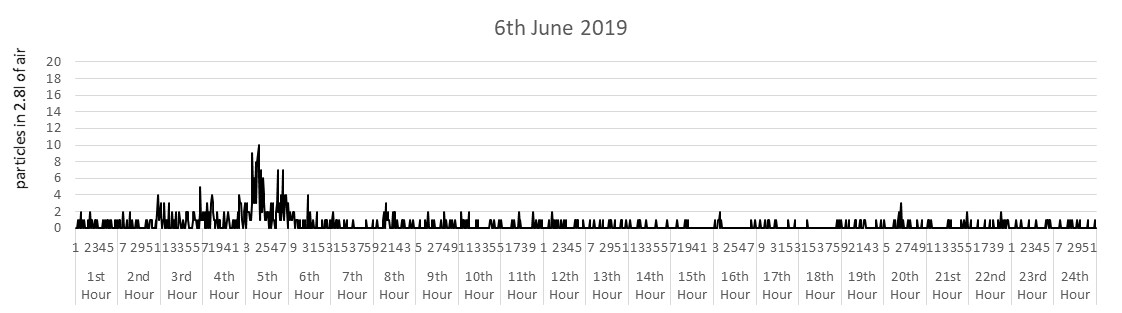* |
| *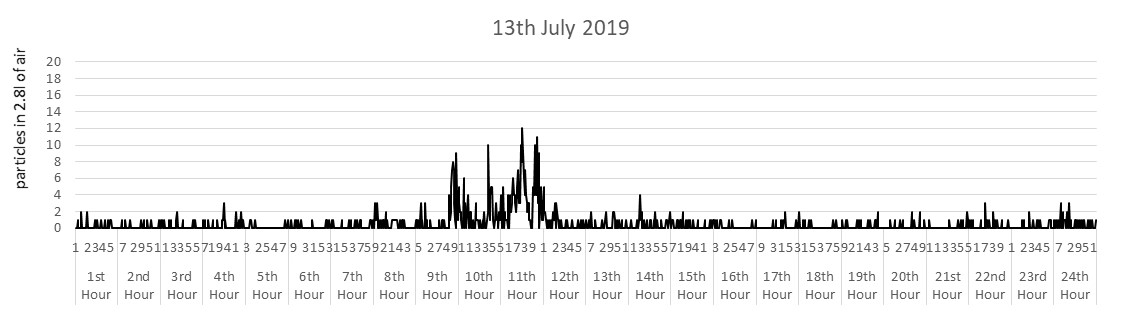* |
| *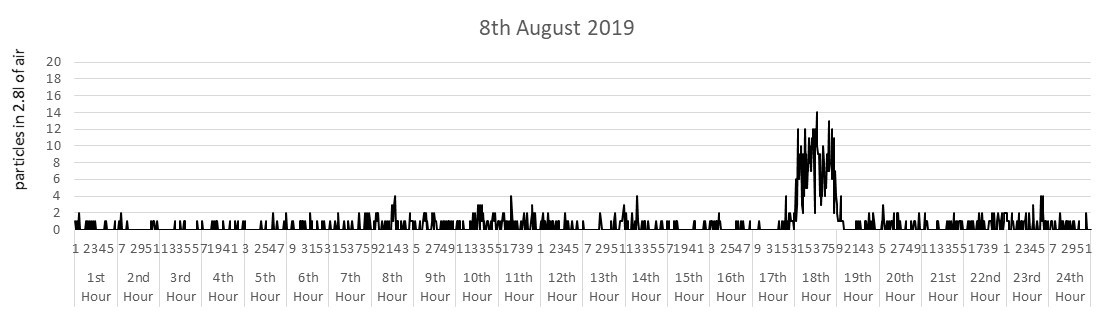* |
| *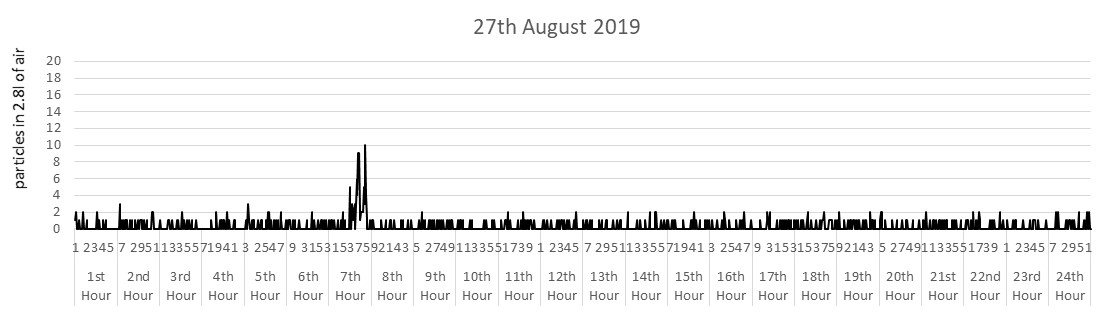* |
| *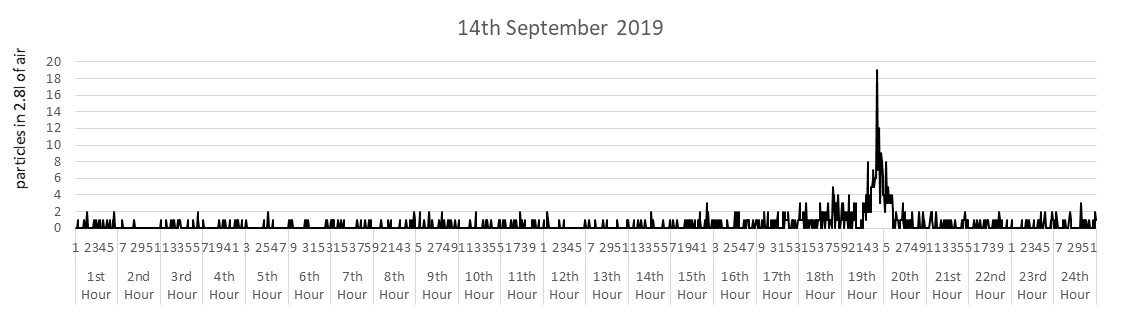* |
| 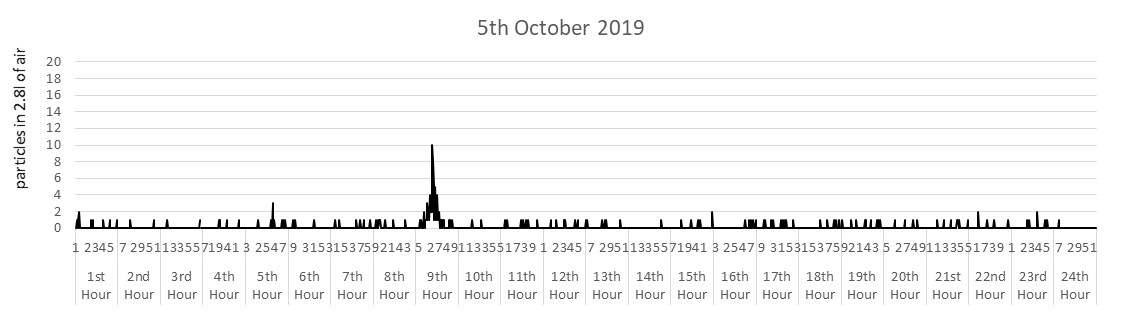 |
| Supplementary Figure S3. 1-minute starch grains detections from Rapid-E bioaerosol monitor on selected days when large quantity of starch is recorded. Data in UTC. |

| 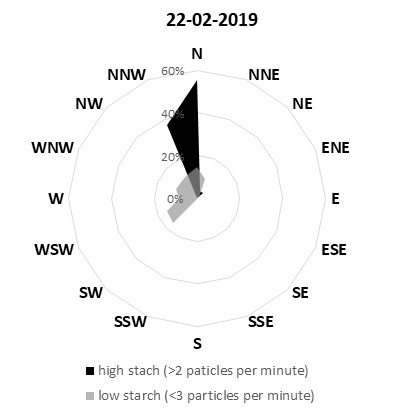 | 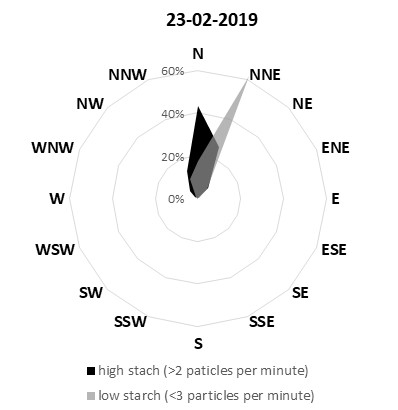 | 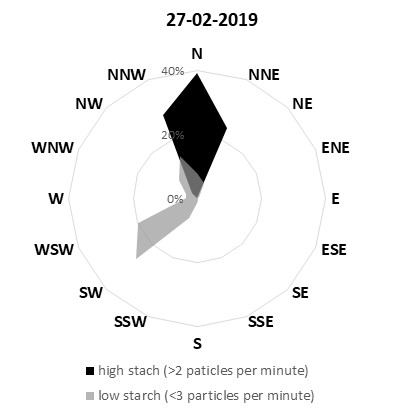 | 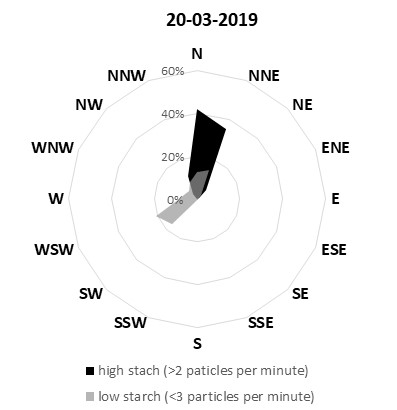 | 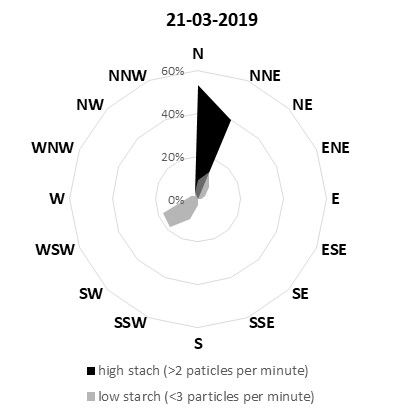 |
| --- | --- | --- | --- | --- |
| 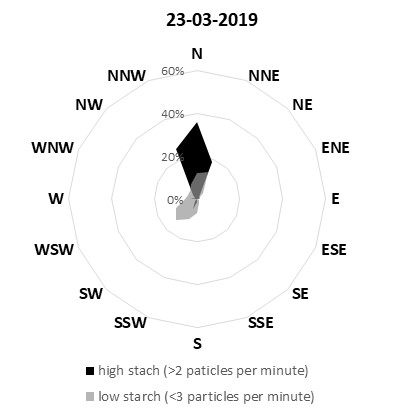 | 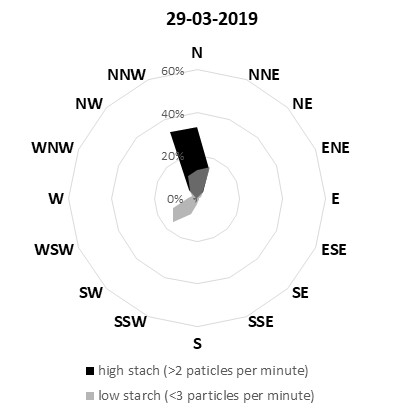 | 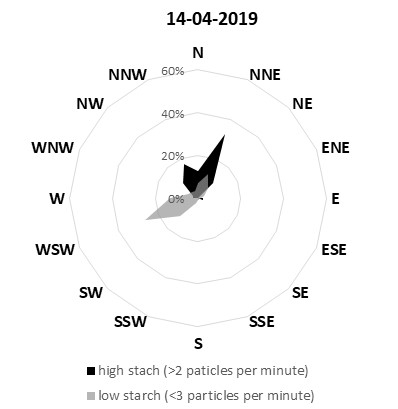 | 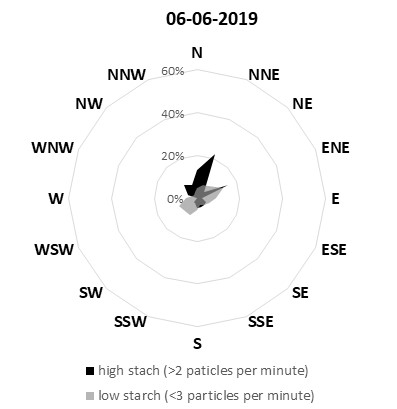 | 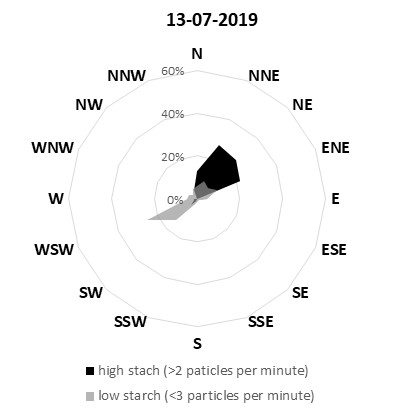 |
| 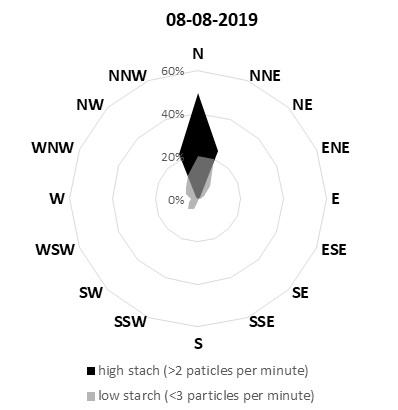 | 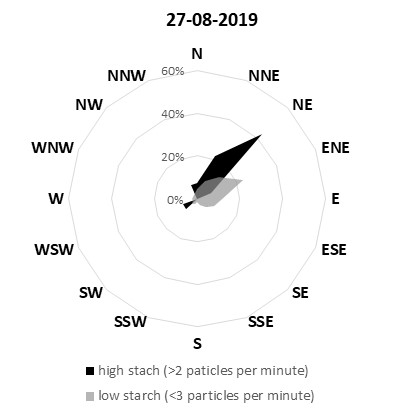 | 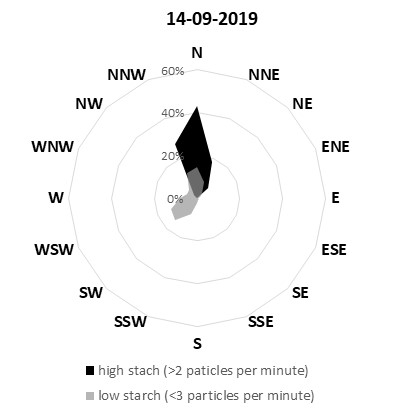 | 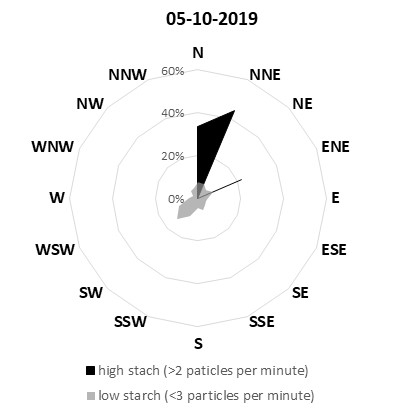 | Supplementary Figure S4. Average wind direction for minutes with low (< 3) and high (> 2) starch counts. |

| 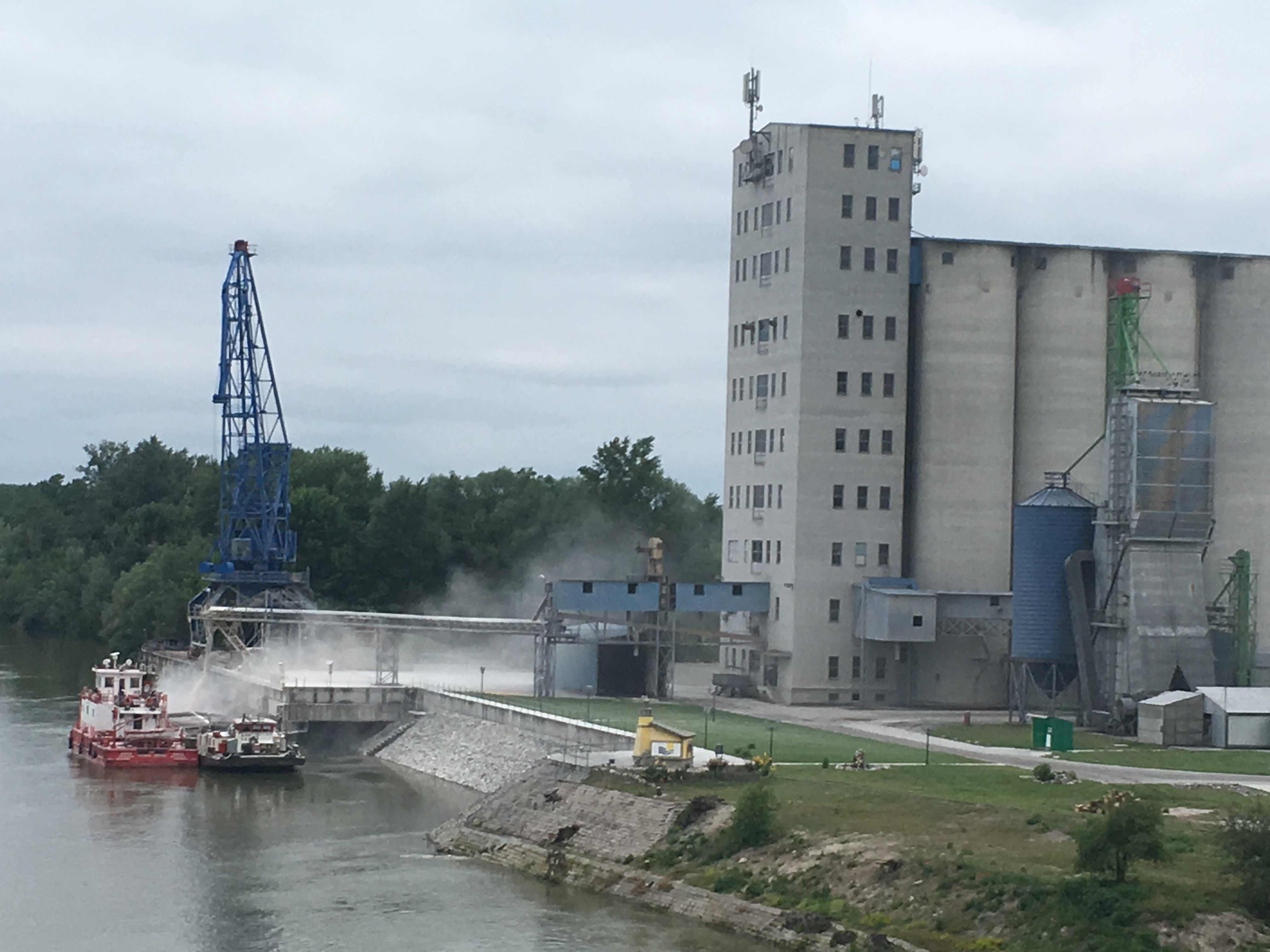 |
| --- |
| Supplementary Figure S5. Flour dust emission during grain unloading from storage towers at port Bogojevo (Serbia) on 16 May 2019. These towers correspond to storage towers in vicinity of each aerobiological station. |

| (A)  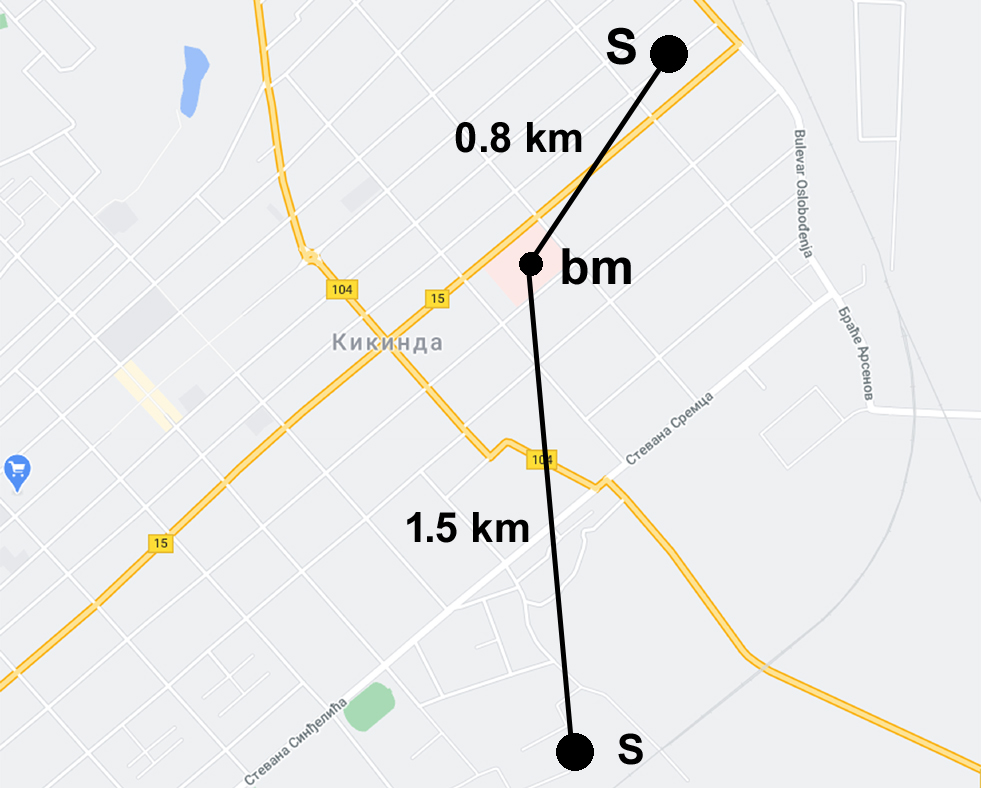 | 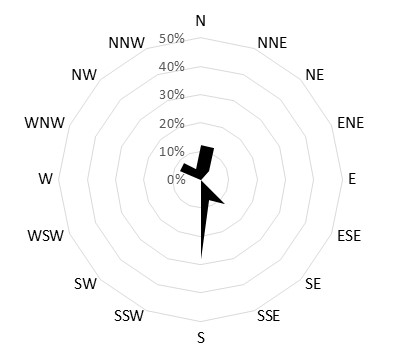 |
| --- | --- |
| (B)  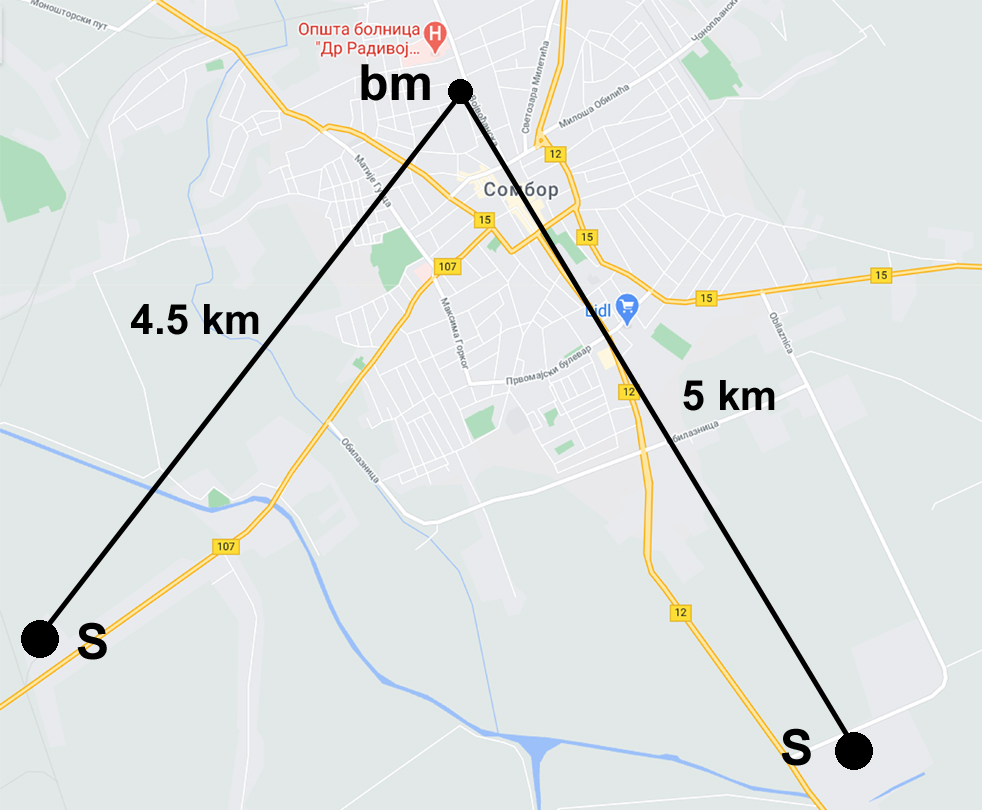 | 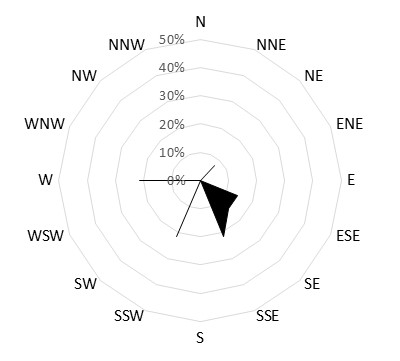 |
| (C)  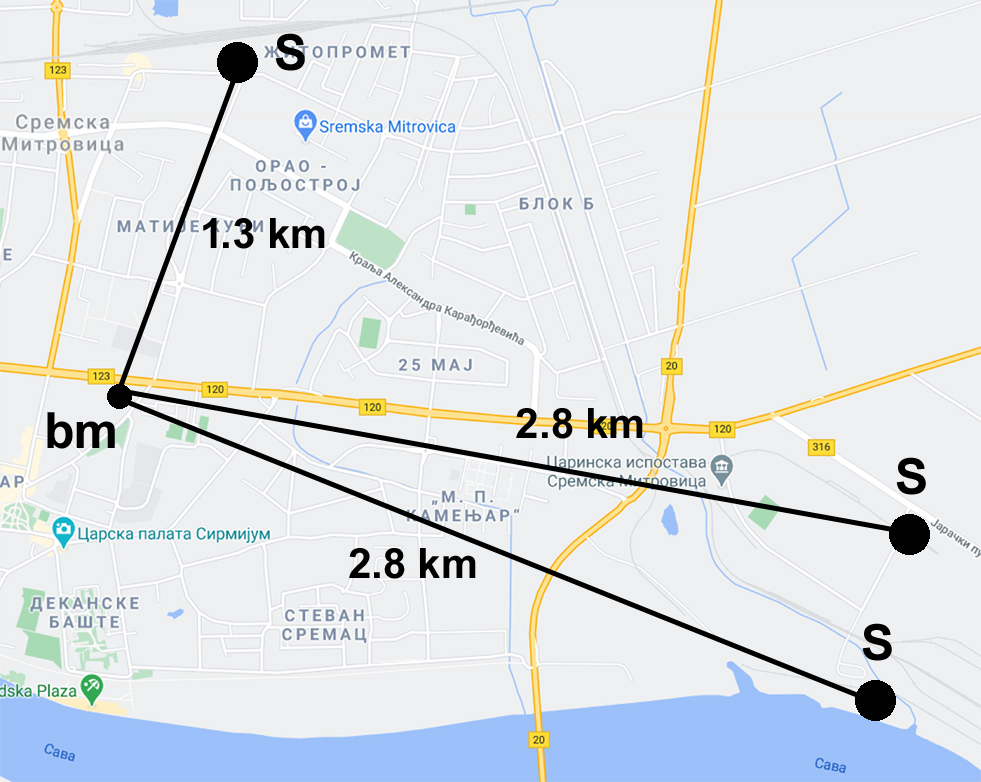 | 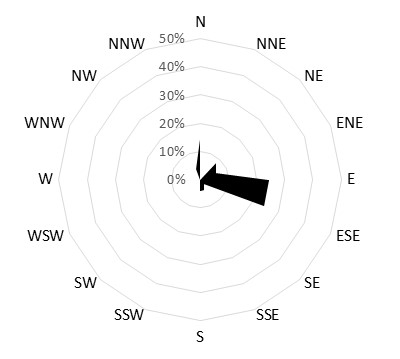 |
| (D)  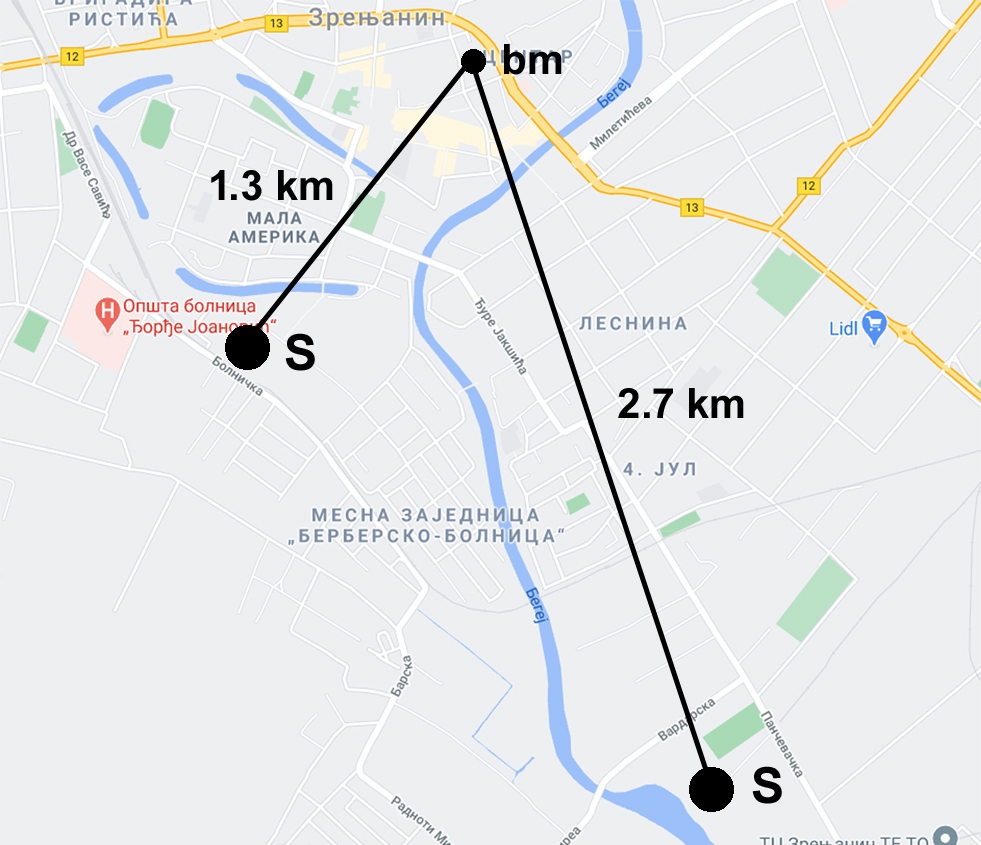 | 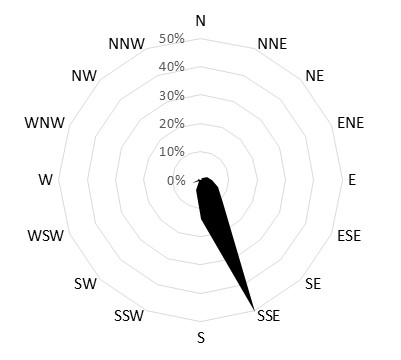 |
| (E)  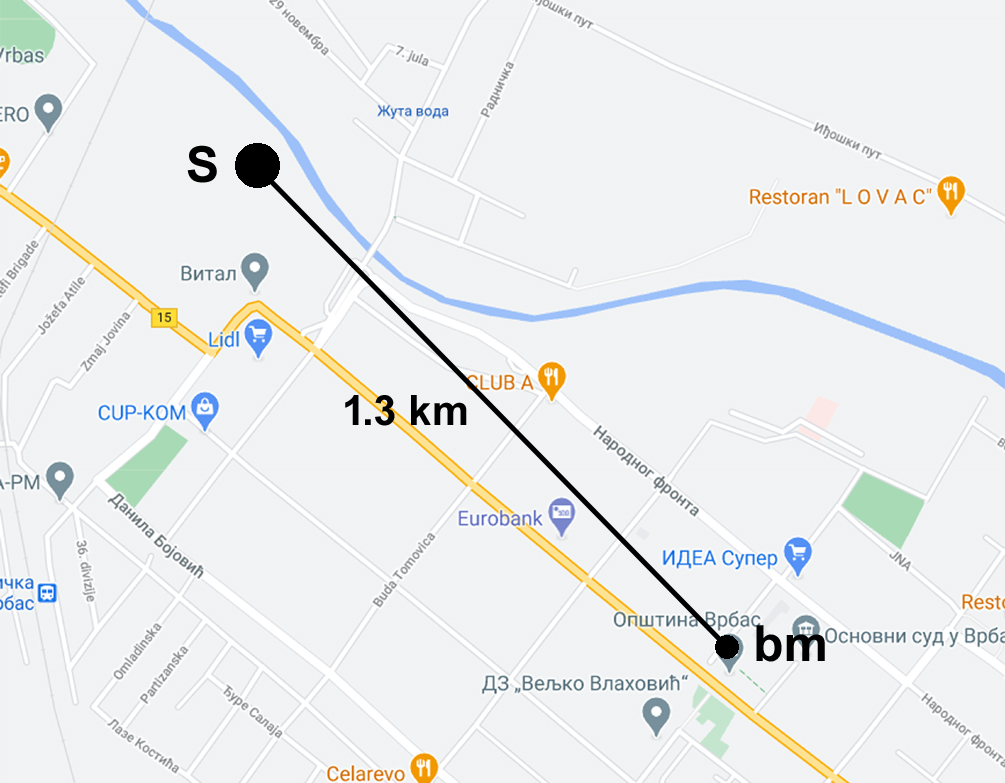 | WIND DATA NOT AVAILABLE |
| Supplementary Figure S6. (A) Kikinda, (B) Sombor, (C) Sremska Mitrovica, (D) Zrenjanin and (E) Vrbas. Right-hand side pannels present wind roses based on wind direction for hours when notable amount of starch particles were recorded, left-hand pannels display location of bioaerosol monitoring station (bm) and potential starch sources (S) as displayed in Google Maps (https://maps.google.com). Distances are measured by Google Earth (<https://earth.google.com/>) | |

NAMELIST.INPUT

&time_control

run_days = 1

run_hours = 0,

run_minutes = 0,

run_seconds = 0,

start_year = 2019,

start_month = 02,

start_day = 22,

start_hour = 00, 06, 12,

start_minute = 00, 00, 00,

start_second = 00, 00, 00,

end_year = 2019,

end_month = 02,

end_day = 23,

end_hour = 00, 00, 12,

end_minute = 00, 00, 00,

end_second = 00, 00, 00,

interval_seconds = 21600

input_from_file = .true.,.true.,.true.,

history_interval = 60, 180, 60,

frames_per_outfile = 1000, 1000, 1000,

restart = .false.,

restart_interval = 5000,

io_form_history = 2

io_form_restart = 2

io_form_input = 2

io_form_boundary = 2

debug_level = 0

/

&domains

time_step = 60,

time_step_fract_num = 0,

time_step_fract_den = 1,

max_dom = 1,

s_we = 1, 1,

e_we = 151, 133, 94,

s_sn = 1, 1,

e_sn = 151, 81, 91,

e_vert = 30, 30, 30,

p_top_requested = 5000,

num_metgrid_levels = 32,

num_metgrid_soil_levels = 4,

dx = 10000, 2500, 3333.33,

dy = 10000, 2500, 3333.33,

grid_id = 1, 2, 3,

parent_id = 0, 1, 2,

i_parent_start = 1, 10, 30,

j_parent_start = 1, 37, 30,

parent_grid_ratio = 1, 4, 3,

parent_time_step_ratio = 1, 4, 3,

feedback = 1,

smooth_option = 0

/

&physics

mp_physics = 10, 3, 3,

ra_lw_physics = 1, 1, 1,

ra_sw_physics = 1, 1, 1,

radt = 30, 30, 30,

sf_sfclay_physics = 1, 1, 1,

sf_surface_physics = 2, 2, 2,

bl_pbl_physics = 1, 1, 1,

bldt = 0, 0, 0,

cu_physics = 1, 1, 0,

cudt = 5, 5, 5,

isfflx = 1,

ifsnow = 1,

icloud = 1,

surface_input_source = 1,

num_soil_layers = 4,

sf_urban_physics = 0, 0, 0,

prec_acc_dt = 180,

NUM_LAND_CAT = 21,

/

&fdda

/

&dynamics

w_damping = 0,

diff_opt = 1, 1, 1,

km_opt = 4, 4, 4,

diff_6th_opt = 0, 0, 0,

diff_6th_factor = 0.12, 0.12, 0.12,

base_temp = 290.

damp_opt = 0,

zdamp = 5000., 5000., 5000.,

dampcoef = 0.2, 0.2, 0.2

khdif = 0, 0, 0,

kvdif = 0, 0, 0,

non_hydrostatic = .true., .true., .true.,

moist_adv_opt = 1, 1, 1,

scalar_adv_opt = 1, 1, 1,

/

&bdy_control

spec_bdy_width = 5,

spec_zone = 1,

relax_zone = 4,

specified = .true., .false.,.false.,

nested = .false., .true., .true.,

/

&grib2

/

&namelist_quilt

nio_tasks_per_group = 0,

nio_groups = 1,
